# Medial unicompartmental knee arthroplasty: increasingly uniform patient demographics despite differences in surgical volume and usage—a descriptive study of 8,501 cases from the Danish Knee Arthroplasty Registry

**DOI:** 10.1080/17453674.2019.1601834

**Published:** 2019-04-11

**Authors:** Cecilie Henkel, Mette Mikkelsen, Alma B Pedersen, Lasse E Rasmussen, Kirill Gromov, Andrew Price, Anders Troelsen

**Affiliations:** a Department of Orthopaedic Surgery, Clinical Orthopaedic Research Hvidovre (CORH), Copenhagen University Hospital Hvidovre, Denmark;;; b Department of Clinical Epidemiology, Aarhus University Hospital, Denmark;; c Department of Orthopaedic Surgery, Vejle Hospital, Denmark;; d Nuffield Department of Orthopaedic Surgery, Rheumatology and Musculoskeletal Science, Nuffield Orthopaedic Centre, University of Oxford, UK

## Abstract

Background and purpose — Using contemporary indications, up to 50% of patients undergoing knee arthroplasty are eligible for unicompartmental knee arthroplasty (UKA), and lower UKA use likely reflects a restrictive approach to patient selection. Since broader indications have been successfully introduced, and low surgical volume and UKA percentage (usage) are associated with higher revision rates, it is of interest whether the actual use of UKA has changed accordingly. We explored this by assessing time trends in patient demographics and whether these are associated with center UKA volume and usage.

Patients and methods — From the Danish Knee Arthroplasty Registry, we included 8,501 medial UKAs performed for primary osteoarthritis during 2002–2016. Using locally weighted regression, we examined changes—both overall and by center volume and usage (low vs high)—in sex distribution, age, weight, and preoperative American Knee Society Score (AKSS-O).

Results — Over the last 20 years, UKA use in Denmark has been increasing steadily. Age, weight, and proportion of men all increased regardless of volume and usage. AKSS-O showed an initial increase followed by a decrease. In low-usage and low-volume centers, the proportion of women was higher, patients were younger, weighed less, and had higher AKSS-O scores; however, for age and AKSS-O, the groups were converging during the last part of the period.

Interpretation — Characteristics of UKA patients have changed in the last 15 years irrespective of center volume and usage. We found between-group differences for both volume and usage, though with convergence for age and AKSS-O, which suggests an increasingly uniform approach to patient selection.

Unicompartmental knee arthroplasty (UKA) is a viable alternative to total knee arthroplasty (TKA) for patients with pronounced, isolated anteromedial osteoarthritis of the knee, resulting in lower mortality and morbidity (Liddle et al. 2014) as well as better patient-reported outcomes (Liddle et al. 2015a, Burn et al. 2018). However, revision rates are higher for UKA than for TKA (Liddle et al. 2014, Chawla et al. 2017).

Kozinn and Scott (1989) proposed a set of strict contraindications for UKA, leaving just 6% of patients eligible for the procedure (Stern et al. 1993). These have now proven unnecessary (Pandit et al. 2011, Hamilton et al. 2017a), and broadening the indications increases the proportion of patients eligible for UKA to as much as 50% (Willis-Owen et al. 2009). Historically, an increase in UKA use was considered to be problematic as the revision burden was assumed to grow. However, low surgical UKA volume and usage—defined as the total annual number of UKAs, and the percentage of all primary knee arthroplasties that are UKA, respectively—have both been associated with higher revision rates (Liddle et al. 2015b, Liddle et al. 2016, Badawy et al. 2017). Interestingly, Hamilton et al. (2017b) found that the positive effect of high UKA usage was independent of UKA volume. Since higher usage is obtained through more liberal patient selection, these findings suggest that a restrictive approach to patient selection is unnecessary. Therefore, it is of interest whether the practice regarding UKA has changed in accordance with the shift towards wider indications.

To investigate this, we explored time trends in UKA use and patient demographics among UKA patients registered in the Danish Knee Arthroplasty Registry (DKR). In addition, we assessed whether they are associated with center UKA volume and usage.

## Patients and methods

We conducted a descriptive study using data from the Danish Knee Arthroplasty Registry (DKR), which has been collecting pre- and perioperative data on knee arthroplasty procedures performed in Denmark since 1 January 1997 (Pedersen et al. 2012).

### Study population

Our data extraction was done on 4 December 2017 and included all primary medial UKAs reported until this date. Due to possible confounding, only medial UKAs performed for primary osteoarthritis were included in the evaluation of patient characteristics. Procedures from 2017 were excluded in assessment of volume and usage as data were incomplete for this year, which hindered the calculation of meaningful values. Due to a low number of procedures (< 100 annually) during the first 5 years, procedures done in the period 1997–2001 were excluded as well.

### Patient demographics

To describe changes in patient demographics, we included information on sex, age, weight, and preoperative American Knee Society Score (AKSS-O) (Insall et al. 1989). Weight values < 45 kg and > 200 kg were considered registration errors and were treated as missing values.

### Volume and usage

We defined center volume as the total number of medial UKAs performed at the center in 1 calendar year. Likewise, we defined usage as the percentage of all primary knee arthroplasty procedures at the center that were medial UKAs in the given calendar year. Volume and usage were assessed independently for each year, making it possible for centers to change groups from year to year. Upon calculation of center volume and usage, patients were assigned the given values of the center responsible for their procedure, and all subsequent calculations were done at the patient level.

For investigation of volume and usage, both were divided into 2 groups: low and high. For volume, we based the grouping on the distribution of our data, as is often done (Robertsson et al. 2001, Lau et al. 2012, Baker et al. 2013, Badawy et al. 2017). We made the cut at the median value to obtain 2 groups of approximately equal size, thus making centers performing < 52 UKAs annually low volume and centers performing ≥ 52 UKAs annually high volume. For usage, we based our categorization on the works of Liddle et al. (2015b) and Hamilton et al. (2017b), which show that a usage of ≥ 20% yields acceptable results. Hence, centers with a usage of < 20% were categorized as low usage and centers with a usage of ≥ 20% were categorized as high usage regardless of their volume.

### Statistics

Unless otherwise specified, reported values are mean (SD). In addressing missing values, we chose to omit the patients for the variable(s) at issue but included them for all other investigations.

All graphical explorations relating to patient characteristics were performed using locally weighted regression (Cleveland 1979). For each of the 4 variables, both an overall and separate locally estimated scatterplot smoothing (LOESS) curves for the 4 volume and usage groups were fitted. With the exception of weight, all variables were normally distributed. In a subgroup assessment, we excluded all bilateral procedures and repeated the LOESS curves. Calculations and graphs were made in R (version 3.3.2, the R Foundation for Statistical Computing, Vienna, Austria).

### Ethics, funding, and potential conflicts of interest

The study was approved by the Danish Data Protection Agency (J No 2012-58-0004). No external funding was received, and the authors declare no conflicts of interest.

## Results

**Figure 1.**
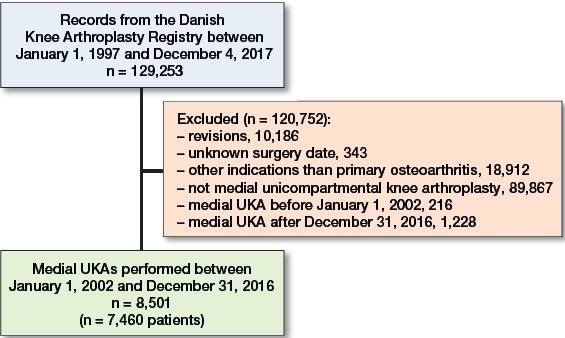
Selection of the final study population.

Selection of the final study population is mapped out in Figure 1 and patient characteristics are summarized in Table 1. Data completion was high, with a complete set for sex and age, 310 (3.1%) missing values for weight, and 178 (1.8%) missing values for AKSS-O.

**Table 1.** Characteristics of the final study population. Values are mean (standard deviation) unless otherwise specified

Number of patients	8,501
Low volume	4,097
High volume	4,404
Low usage	3,313
High usage	5,188
Number of women^a^	4,697 (55)
Low volume	2,274 (56)
High volume	2,423 (55)
Low usage	1,885 (57)
High usage	2,812 (54)
Age (years)	65.0 (9.4)
Low volume	64.2 (9.6)
High volume	65.8 (9.2)
Low usage	64.5 (9.6)
High usage	65.3 (9.3)
Weight (kg)^b^	82 (45–190)
Low volume	82 (45–188)
High volume	83 (45–190)
Low usage	82 (45–188)
High usage	83 (45–190)
Knee score (AKSS-O)	42 (14)
Low volume	44 (15)
High volume	40 (13)
Low usage	43 (15)
High usage	41 (14)

Overall values and grouped by center volume (low < 52 per year, high ≥ 52 per year) and usage (low < 20%, high ≥ 20%).

a Frequency (%)

b Median (range)

**Figure 2.**
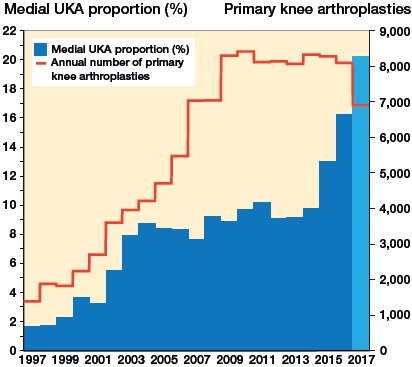
Annual use of medial UKAs. The national percentage of all primary knee arthroplasties accounted for by medial unicompartmental arthroplasties (UKAs) each year and the annual numbers of all primary arthroplasties. The numbers account for all registered knee arthroplasty procedures except revisions. Note that the dataset contains only procedures up to December 4, 2017, which is why the numbers for 2017 should be interpreted with caution.

The use of UKA in Denmark has been steadily increasing since 1997 (Figure 2). The median center volume was 52 (1–234) and the median center usage 22% (0.2–100), both with time trends analogous to the overall increase in use. In 1997, 10 centers reported UKA procedures, which increased steadily to 17 in 2002 and further to 25 centers in 2006 (Table 2). From 2006 to 2008, there was a rapid increase to 35 centers, which was matched by an increase of 10 low-volume centers, but only by 2 additional low-usage centers. From 2008 to 2016, the total number of reporting centers decreased to 25, primarily caused by a decrease in low-volume and low-usage centers.

**Table 2.** Number of centers (C) performing medial unicompartmental arthroplasties (UKAs) each year and the total number of patients (P)^a^

	Low volume		High volume		Low usage		High usage		Total	
	C	P	C	P	C	P	C	P	C	P
1997	10	23	0	0	10	23	0	0	10	23
1998	15	32	0	0	14	23	1	9	15	32
1999	11	41	0	0	9	17	2	24	11	41
2000	13	81	0	0	11	57	2	24	13	81
2001	13	87	0	0	12	39	1	48	13	87
2002	16	132	1	65	13	48	4	149	17	197
2003	20	188	2	125	18	150	4	163	22	313
2004	23	283	1	85	19	163	5	205	24	368
2005	26	261	2	132	24	194	4	199	28	393
2006	22	245	3	209	20	233	5	221	25	454
2007	27	348	3	187	22	317	8	218	30	535
2008	32	469	3	178	22	269	13	378	35	647
2009	29	344	5	394	22	367	12	371	34	738
2010	31	405	5	411	25	481	11	335	36	816
2011	25	217	7	612	21	315	11	514	32	829
2012	24	268	5	468	19	240	10	496	29	736
2013	18	262	4	476	15	278	7	460	22	738
2014	20	258	5	556	15	346	10	468	25	814
2015	17	289	5	790	12	321	10	758	22	1,079
2016	17	263	8	1,054	15	373	10	944	25	1,317

a Both grouped by center volume (low < 52 per year, high ≥ 52 per year) and usage (low < 20%, high ≥ 20%).

The 8,501 procedures in 2002–2016 were performed in 7,460 patients, and subgroup assessment with exclusion of bilateral procedures showed similar distribution of the demographic variables. The most common implant type was the mobile-bearing Oxford knee (91%; n = 7,693), followed by the fixed-bearing implants ZUK (2%; n = 194) and Link (2%; n = 154).

### Patient characteristics

#### Sex

The proportion of females has been steadily decreasing from 67% (n = 115) in 2002 to 55% (n = 622) in 2016. The same pattern was seen in both low- and high-volume centers. Low-volume centers had a lower proportion of females in the years 2002–2007 and 2013–2016 but a higher proportion in 2008–2012. Both usage groups also showed an overall decreasing trend, and low-usage centers had a higher proportion of females throughout the study period.

#### Age

**Figure 3.**
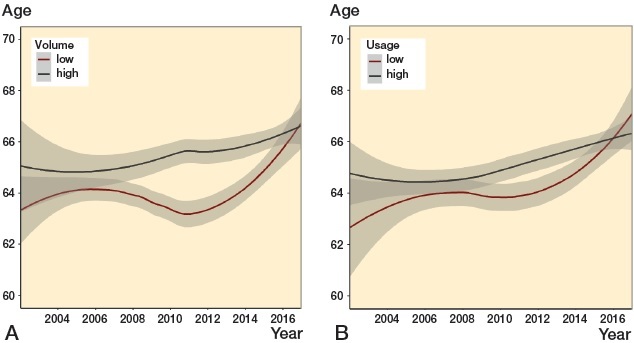
Age. LOESS curves (with 95% confidence intervals) depicting time trends in the age of UKA patients in: A: centers with low (< 52 per year) and high (≥ 52 per year) volume. B: centers with low (< 20%) and high (≥ 20%) usage.

The age has been increasing, from 64 years (9.6) in 2002 to 66 years (9.2) in 2016. Both volume groups shared this tendency, but patients from low-volume centers were generally younger. In 2007–2011 the low-volume centers expressed a temporary decrease, which resulted in a more pronounced difference between the groups. The decrease was followed by a substantial increase, eliminating the difference between the groups by 2016. The usage groups showed a similar pattern, though less pronounced (Figure 3). In 2016 the groups seem to have switched, presenting a higher age for the low-usage group.

#### Weight

The weight has generally shown an increasing trend, from a median weight of 82 kg (47–150) in 2002 to 85 kg (47–186) in 2016. Though the males were generally heavier, there was no marked difference in the overall tendencies between the sexes. Both volume groups also shared the overall increasing tendency. The low-volume group had a lower weight in 2005–2015 but higher both before and after this period. For usage, both groups expressed the overall increasing tendency as well, but with lower weights in patients from low-usage centers during the entire period.

#### Knee score (AKSS-O)

**Figure 4.**
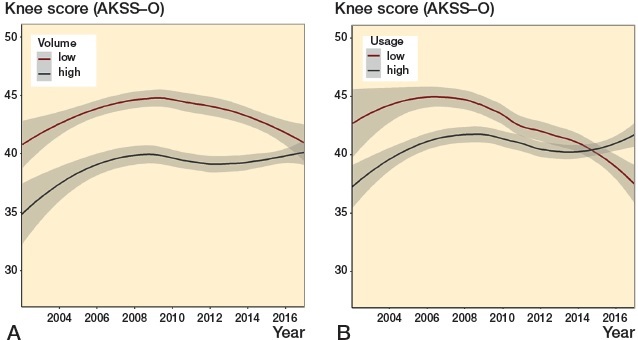
Knee score (AKSS-O). LOESS curves (with 95% confidence intervals) depicting time trends in preoperative knee score (AKSS-O) for UKA patients in: A: centers with low (< 52 per year) and high (≥ 52 per year) volume. B: centers with low (< 20%) and high (≥ 20%) usage.

The general trend in knee score was bell-shaped with an initial increase from 39 (15) in 2002 to its peak at 44 (16) in 2006, followed by a decrease to 40 (13) in 2016. The low-volume group had a higher score throughout the period, but the trends were converging and in 2016 the difference was minor. Low-usage centers had higher scores until 2014, where the scores for high-usage centers started increasing while the scores for low-usage centers continued to decrease (Figure 4).

## Discussion

In our national registry-based study we found that the use of UKA has increased substantially in the past 20 years accompanied by a change in patient characteristics. Patient characteristics differed with center volume and usage, though recent converging trends were noted.

The total number of medial UKAs has increased steadily since 1997 but most markedly in 2015–2016. The total number of TKAs has also increased, though with a notable drop in 2015–2016. Looking at the annual sums of medial UKAs and TKAs during 2011–2016, they have generally been stable in this period. Therefore, the drop in number of TKAs in 2015–2016 may be explained by the simultaneous increase in medial UKAs, thus resulting in the considerable increase in UKA percentage during the last few years. The recent stagnation in the total annual number of knee arthroplasties in Denmark might be in contrast to other countries, e.g. Sweden where the number of procedures is still increasing (SKAR 2018). However, in Denmark as well as in Sweden, the incidence of knee arthroplasty was increasing until levelling off in 2009. In 2011–2016, the incidences were stable, in Denmark at an average of 147/100,000 inhabitants (DKR 2017) and in Sweden 137/100,000 inhabitants (SKAR 2018). Hence, the different trends in annual number of procedures between the countries do not necessarily reflect differences related directly to arthroplasty surgery. External factors such as population growth rate might also play a part, and hence, direct comparison between the countries contains possible pitfalls. Regarding stagnation of the number of procedures in Denmark, this followed a 4-fold increase from 2000 to 2009. This increase was possibly facilitated by the introduction of fast-track programs, resulting in shorter hospital stays and thereby increased capacity. In the later years, an increasing focus on nonoperative treatment, e.g., the nationwide implementation of the initiative Good Life with osteoArthritis in Denmark (GLA:D) (Skou and Roos 2017), may have led to postponement of surgery. Indicative of this is the peak in number of knee arthroplasties in 2010, coincident with the lowest mean age in the history of the registry (DKR 2017), which was followed by an increasing mean age concurrent with the stabilization in the number of procedures (DKR 2017). Altogether, the recent stagnation in number of knee arthroplasties could indicate that we have reached an appropriate level of treatment with an adequate capacity.

In Sweden, the pattern of UKA use has been markedly different from that in Denmark (SKAR 2015, SKAR 2018). This may reflect controversy in how to weigh the advantages against the risk of UKAs compared with TKAs. From the 1990s until 2014, the use of UKA in Sweden was reduced dramatically, and in 2014 the Swedish medial UKA percentage was just 3.4%. However, from 2014 to 2017 the medial UKA percentage in Sweden more than doubled to 7.7%. In 2014, 53% of the UKA implants in Sweden were Oxford and 40% were ZUK or Link. In 2017, this distribution changed to 66% Oxford and 23% ZUK or Link. With an increasing use of UKA and a higher proportion of Oxford implants at the expense of fixed-bearing implant types, the recent trends in Sweden might indicate a change equivalent to that we have seen in Denmark.

In our study population, the proportion of female patients has been decreasing. This is similar to—though more pronounced than—the trend in the overall population of knee arthroplasty patients in Denmark (DKR 2017). A possible explanation for the greater decrease is reporting of higher revision rates among female compared with male UKA patients in the national registry (DKR 2017). Weight trends were increasing for both male and female patients, and the magnitude of the increments is comparable to that of the general population (Christensen et al. 2016). Thus, it is plausible that the weight trend we observe is mainly a reflection of changing demographics in the Danish population along with an increasing proportion of male patients. This is further supported by the analogous time patterns for sex and weight when grouped by volume and usage.

Regarding age, the increasing trend we observed differs from the trend in the general knee arthroplasty population, which has been rather stable at 67–68 years (DKR 2017). Though UKA patients are still younger than the overall knee arthroplasty population, the difference is diminishing. This may be indicative of increasing uniformity in patient selection between UKAs and TKAs. Previous studies have reported that patients who received a UKA from a low-volume (Liddle et al. 2016) or a low-usage (Liddle et al. 2015b, Hamilton et al. 2017b) surgeon tended to be younger, which is in line with our findings. It has been hypothesized that this could be associated with a tendency to perform UKA in patients with earlier-stage disease (Hamilton et al. 2017b, Murray and Parkinson 2018). If this is indeed the case, it could explain the higher knee scores among patients from low-volume and low-usage centers. This hypothesis is further supported by a study from Jones et al. (2016) reporting that patients with early radiographic osteoarthritis were younger than patients receiving UKAs for bone-on-bone osteoarthritis. Overall for the study period, the knee score in our study population is approximately 7 points higher than reported for knee arthroplasty patients in the latest annual report from DKR (DKR 2017). This is not surprising as the indications for the Oxford implant include a flexion deformity less than 15 degrees and an intact anterior cruciate ligament (Goodfellow 2011), both factors that are represented in the knee score (Insall et al. 1989). Notably, the bell-shaped time trend is also detectable in the overall knee arthroplasty population (DKR 2017), and therefore it is unlikely that the pattern can be attributed to factors relating uniquely to the UKA procedure. It is noteworthy that reporting of procedures to the DKR became mandatory in 2006 (Pedersen et al. 2012), which resulted in an increase in completeness in the registry from 82% in 2005 to 93% in 2007 (DKR 2017). This calls into question whether the trends seen before 2006 are distorted by reporting bias.

When comparing the effects of volume with the effects of usage, it is notable that the time trends are generally similar for the low- and high-usage groups while the volume groups show more varying patterns. For sex, age, and weight, the low-volume centers tend to deviate from the overall trend around 2007. This is coincident with the increase in the total number of centers performing UKA from 25 in 2006 to 35 in 2008. Data from the DKR annual report show that, in the same period, 14 new private knee arthroplasty centers appeared (DKR 2017). It is plausible that patient demographics differ in these new private centers; and as the increase in the total number of centers was accompanied by the occurrence of 10 additional low-volume centers but only 2 additional low-usage centers, this could explain the more fluctuating trends in the volume groups compared with the usage groups. In 2007 there was a change in legislation, giving patients the right to have their tax-financed surgery performed at a private center if the waiting time at their public hospital exceeded one month. This is a plausible explanation for the aforementioned changes and, as such, the changes in patient characteristics could represent either surgeon proclivity or selection bias in the patients making use of this new opportunity.

As our study is based on an unselected population from a national registry, the external validity is generally high. However, as discussed above, the trends we see are results of complex interactions of changes in demographics and structure as well as factors relating to centers and surgeons, possibly including surgical technique. This complicates interpretation of our findings and might impede generalizability. Another limitation of the study is the categorization of volume and usage. Procedures in DKR are linked to centers and not surgeons, which is why our categorization is center-based. Baker et al. (2013) demonstrated that both surgeon and center volumes were associated with revision but, in addition, that surgeon volume was the more valuable measure of the 2. Hence, the operating surgeon is a possible confounder in our study. For both volume and usage, it is questionable whether our cut-off values are clinically relevant, and choosing other cut-off values might affect the results. In addition to this, the calculation of volume and usage was done for each calendar year. This ensured that centers would not be at a disadvantage if they either started or ceased performing UKA during the study period. But it also means that the volume or usage group does not necessarily express a center’s level of experience altogether.

Our findings indicate how external factors can influence our data, emphasizing that aspects such as structural changes should always be considered when interpreting data. Volume seemed to be more vulnerable to this, and therefore we suggest that usage is a more robust measure and should be preferred in future research.

In conclusion, there has been a considerable increase in use of UKA in Denmark, accompanied by a shift in patient demographics toward an older population with an increasing proportion of males, higher weights, and lower knee scores. Patient characteristics differed with center volume and usage, though for age and knee score the groups were converging. This suggests an increasingly uniform approach to patient selection in accordance with the more permissive view on candidacy.
